# Edinburgh Royal Infirmary

**Published:** 1830-11

**Authors:** 


### EDINBURGH ROYAL INFIRMARY.

### *Strychnia in Amaurosis.**

Case I. Peter Hamilton, ætat. twenty-two, an iron-founder, admitted 16th June, 1829, can only distinguish light from darkness. Both pupils are much dilated, the right more than the left. The iris in both is sensible to the stimulus of light. The eyes are clear, and, with the exception of a slight squint, present a natural appearance. This state of vision has continued two years. His account of its commencement is as follows:

Having been for some years daily working under exposure to the heat and light of an iron-founder's furnace, he became affected with indistinctness of vision, accompanied with flashes of light when looking at minute objects, or when stooping. This indistinctness became gradually more and more obscure for fifteen months. At the end of this time he could only distinguish light from darkness, and has remained in that state nine months. His general health had all along been quite good.

17th. The temples were shaved and blistered, and one eighth of a grain of strychnine dusted the following day on each side.

23d June, (6th day.) Within the last week a blister has been, twice in succession, applied to each temple, and to the raw surfaces, first one eighth, then one fourth, and today half a grain of the powder of strychnia. The pupils are less dilated, and the iris readily contractile; strabismus almost gone; tongue rather foul; bowels open.

25th. Can today distinguish colours pretty readily, especially with the left eye, the iris of which is less sensible than that of the right. Half a grain was applied to each temple.

26th. Still continues to improve, and can distinguish yellow

* Remarks on the Treatment of Amaurosis, &c. by Thomas Shortt, M.D. F.R.S.E. &c. (Edinburgh Med. and Surg. Journal, October.)

Dr. Shortt considers strychnine to act purely as a stimulant either of the nervous matter of the nerves, or of their capillary system, and therefore to be successfully employed only in amaurotic cases which depend on paralysis of the optic nerve and retina, or in cases of congestive amaurosis from pressure on these parts, with a loaded and inactive state of the minute vessels of the neurilematic envelope. As these affections are often ascertained with difficulty, cases may be viewed as such, though arising from structural derangements of the interior of the organ of vision. Mr. Liston, Mr. Guthrie, and other surgeons, have also published cases which show the efficacy of strychnine in amaurosis when it depends upon paralysis or congestion. Dr. Shortt is of opinion that in such cases the beneficial operation of strychnine is considerably aided by the previous use of mercury. He cannot positively say whether the effects of strychnine are lasting, but he believes it in most cases to be so, if properly used. In no case in which he has tried it, have injurious effects arisen. In delicate persons, or where the system is affected by mercury, Dr. S. adds, that the dose of strychnine should not be more at first than a quarter of a grain, increasing it daily until it produces sensible effects on the constitution, as headach, pricking pains over the body, or tremors; it must then be discontinued, and, on resuming it, the dose should always be considerably reduced.

and red colours; some headach, and tongue much loaded and white. Three fourths of a grain to each surface; a cathartic mixture.

27th. Less headach; sight considerably improved, for he can distinguish print from writing. One grain applied to each surface.

28th. Vision more distinct. Had an additional grain and a quarter yesterday. Had one and a half on each surface.

July 2d, (15th day.) Had one grain and three quarters on the 30th. On the 1st, an attack of rigors, debility, sickness, vertigo, and headach, which are now gone, but feels weak. Can now clearly distinguish objects placed at the distance of some paces, and reads easily the hour upon a watch by evening twilight; iris of both eyes quite sensible. Intermit strychnine.

4d. Sight still further improved. Renew the blisters, and one fourth grain of the powder.

13th. Can now distinguish objects clearly at considerable distances. Pupils continue more contracted, although less than naturally. Strychnia, from one fourth to three fourths of a grain, has been applied as before.

26th. Strychnia has not been applied since last report, from a sensation of violent heat over the skin.

August 4th. Since the last report two grains have been applied to each temple, without any obvious effect; but improvement in vision continues.

16th. Has had two grains on each temple for eight days. Repeat blisters, and apply two and a half grains to each surface.

September 8th, (79th day.) Since the last report he has been applying the strychnia every day, from two to three and a half grains on each temple, without any constitutional effect, but with continued improvement in his sight.

There were some days of intermission, when the blisters were obliged to be renewed. Yesterday he left the Infirmary, and attempted to work; but finding that the act of stooping occasioned dimness of sight, he returned the next day, and resumed the strychnine, to the extent of three grains on each temple, and continued gr. ijss. to the 13th. It was then omitted, and on the 31st, when he could see perfectly, he was ordered to apply the vapour of ammonia for a few days. The eyes appeared quite natural, the squinting gone; and he was enabled to tell the time upon the Tron church clock from the Infirmary windows, at the distance of 300 yards.

Case II. Andrew Drummond, æt. thirty-four, a ship-carpenter, admitted October 2d, 1829. This patient can only distinguish light from darkness, a window from the wall. The pupils of both eyes rather contracted, and there is but little sensibility of the iris. In the left eye there is a ring of opaque substance, the remains of a cataract, the centre of which was removed by an operation; but although the light was thereby admitted, he was still unable to see. This ring can only be observed when the pupil is dilated. This state of vision, which, with little change, has continued six years, commenced as follows.

He had been employed as a flaxdresser six months before he was attacked with fever at Dundee. This was about six years ago. But some weeks previous to this attack, dimness of sight had gradually come on; and then, during the dilirium of fever, his sight was totally lost, and likewise his hearing. During his convalescence he regained his hearing, but remained quite blind; sometimes, however, distinguishing light from darkness. A year after this he was admitted into the Infirmary, had the centre of the cataract removed from the left eye, was blistered, and had setons both in the nucha and in the inside of the arms; but he left the hospital without receiving any benefit to his sight. Two years ago he returned to the house, underwent medical treatment, took mercury, renewed his blisters and setons, and left the house again without any distinct improvement, but only thinks he saw a little more clearly. He has, since his fever, enjoyed good general health.

Oct. 3d. A blister having been applied to the right temple, yesterday one fourth of a grain of strychnia was dusted upon the raw surface; and states that he saw a little with the right eye about six hours after the application, and that almost immediately after he felt a shooting pain across his forehead, but no other sensation. One grain to be applied.

5th. Says he did not feel the application of the whole grain so much as the first quarter, but thinks his sight still improved. Full diet. Blisters renewed.

7th, (5th day.) One grain since last report. Can now distinguish large from small letters; some vertigo last night. One and a quarter grain, and Aqua Ammoniæ.

10th. Since last report a fresh blister and three grains have been applied as before. Sees the Tron church steeple from the window, and distinguishes the colours of the ward, which he could not do yesterday. One grain as before, and a cathartic mixture.

13th. Since the 8th, two and a half grains have been applied. Noticed yesterday with his left eye the bars of the window. Two blisters since applied have risen well. Strychnia omitted yesterday, and no further improvement in sight. Apply one and a half grains.

18th. Five grains since the 13th, and a renewal of the blisters. Complains of the light affecting his eyes, which he had not experienced before.

22d. Three grains since the 18th, with alternate blisters on each temple. Can today see men walking at a distance, which he could not do yesterday.

Nov. 7th, Since the 22d, nineteen and a half grains have been applied, at most two and a half grains at once; his sight gradually improving, and the intolerance of light removed entirely. Strychnia has been intermitted, and his appetite improved by the tincture of calumba. He can now see (Nov. 11th,) to read the hour upon a common watch; but he cannot yet distinguish small print.

He continued in hospital till about the end of November, during which time his sight was still further improved.

